# Neutrophils Orchestrate the Periodontal Pocket

**DOI:** 10.3389/fimmu.2021.788766

**Published:** 2021-11-24

**Authors:** Ljubomir Vitkov, Luis E. Muñoz, Janina Schoen, Jasmin Knopf, Christine Schauer, Bernd Minnich, Martin Herrmann, Matthias Hannig

**Affiliations:** ^1^ Vascular & Exercise Biology Unit, Department of Biosciences, University of Salzburg, Salzburg, Austria; ^2^ Clinic of Operative Dentistry, Periodontology and Preventive Dentistry, Saarland University, Homburg, Germany; ^3^ Department of Internal Medicine 3 - Rheumatology and Immunology, Friedrich-Alexander-University Erlangen-Nürnberg (FAU) and Universitätsklinikum Erlangen, Erlangen, Germany; ^4^ Deutsches Zentrum für Immuntherapie (DZI), Friedrich-Alexander-University Erlangen-Nürnberg and Universitätsklinikum Erlangen, Erlangen, Germany

**Keywords:** dysbiosis, dysregulated immunity, NET formation, caspase 4, caspase 11, bacterial membrane vesicles, outer membrane vesicles

## Abstract

The subgingival biofilm attached to tooth surfaces triggers and maintains periodontitis. Previously, late-onset periodontitis has been considered a consequence of dysbiosis and a resultant polymicrobial disruption of host homeostasis. However, a multitude of studies did not show “healthy” oral microbiota pattern, but a high diversity depending on culture, diets, regional differences, age, social state etc. These findings relativise the aetiological role of the dysbiosis in periodontitis. Furthermore, many late-onset periodontitis traits cannot be explained by dysbiosis; e.g. age-relatedness, attenuation by anti-ageing therapy, neutrophil hyper-responsiveness, and microbiota shifting by dysregulated immunity, yet point to the crucial role of dysregulated immunity and neutrophils in particular. Furthermore, patients with neutropenia and neutrophil defects inevitably develop early-onset periodontitis. Intra-gingivally injecting lipopolysaccharide (LPS) alone causes an exaggerated neutrophil response sufficient to precipitate experimental periodontitis. Vice versa to the surplus of LPS, the increased neutrophil responsiveness characteristic for late-onset periodontitis can effectuate gingiva damage likewise. The exaggerated neutrophil extracellular trap (NET) response in late-onset periodontitis is blameable for damage of gingival barrier, its penetration by bacteria and pathogen-associated molecular patterns (PAMPs) as well as stimulation of Th17 cells, resulting in further neutrophil activation. This identifies the dysregulated immunity as the main contributor to periodontal disease.

## Introduction

Periodontitis is a collective term for disorders of the tooth supporting tissues with various aetiologies (1). In general, the most frequently forms of periodontitis can be divided into two main categories triggered by (I) the biofilm attached to the outer tooth surface and (II) by dental pulp necrosis, respectively (1). The latter is also denoted “endodontic-periodontal lesions” (1) and is also a subject of endodontology. The most frequent form of biofilm-triggered periodontitis is the late-onset, formerly referred to as “chronic periodontitis”. The quite common denoting “late-onset” is not a diagnosis, but just tagging the periodontal disorders, which are an age-related condition occurring in humans after the age of 30 (2–4). Late-onset periodontitis is characterised by the formation of a periodontal pocket, a pathological formation of a duct-like space (periodontal crevice) between the pocket epithelium and the subgingival biofilm attached to the tooth root (5). The subgingival biofilm continuously disperses planktonic bacteria, pathogen-associated molecular patterns (PAMPs), and periodontal pathogenic bacteria (6). These afflict the epithelium in order to get internalised and PAMPs impair the epithelial barrier (7). The crevice is filled with the gingival crevicular fluid (GCF) and is where the periodontal pathogens are initially encountered by the first line of host defence, the crevicular neutrophils and the humoral components of innate and adaptive immunity (8). Late-onset periodontitis is characterised by the inability to efficiently control subgingival biofilm (9), damage of the host tooth supporting tissues (5) and transmigration of periodontal pathogens into blood circulation (10). That the late-onset periodontitis is triggered by subgingival dental biofilm is beyond doubt. One may argue that the dental biofilm is also the cause for this disease, or at least the dysbiosis of the subgingival dental biofilm. Currently, some observations relativise the etiological role of subgingival dysbiosis: (I) the age-association of late-onset periodontitis in susceptible individuals and (II) the neutrophil hyper-responsiveness in late-onset periodontitis, (III) the responsiveness of biofilm-induced periodontitis to anti-ageing therapy (11–13) and (IV) a microbiota shift by dysregulated immunity (14, 15).

The aim of this review was to investigate the role of neutrophils in periodontal disease. We consider the factors responsible for resistance, induction, clearance failure and maintenance of this disease and discuss the role of neutrophils in its aetiopathogenesis.

## Periodontitis Pathology

**Figure 1:**
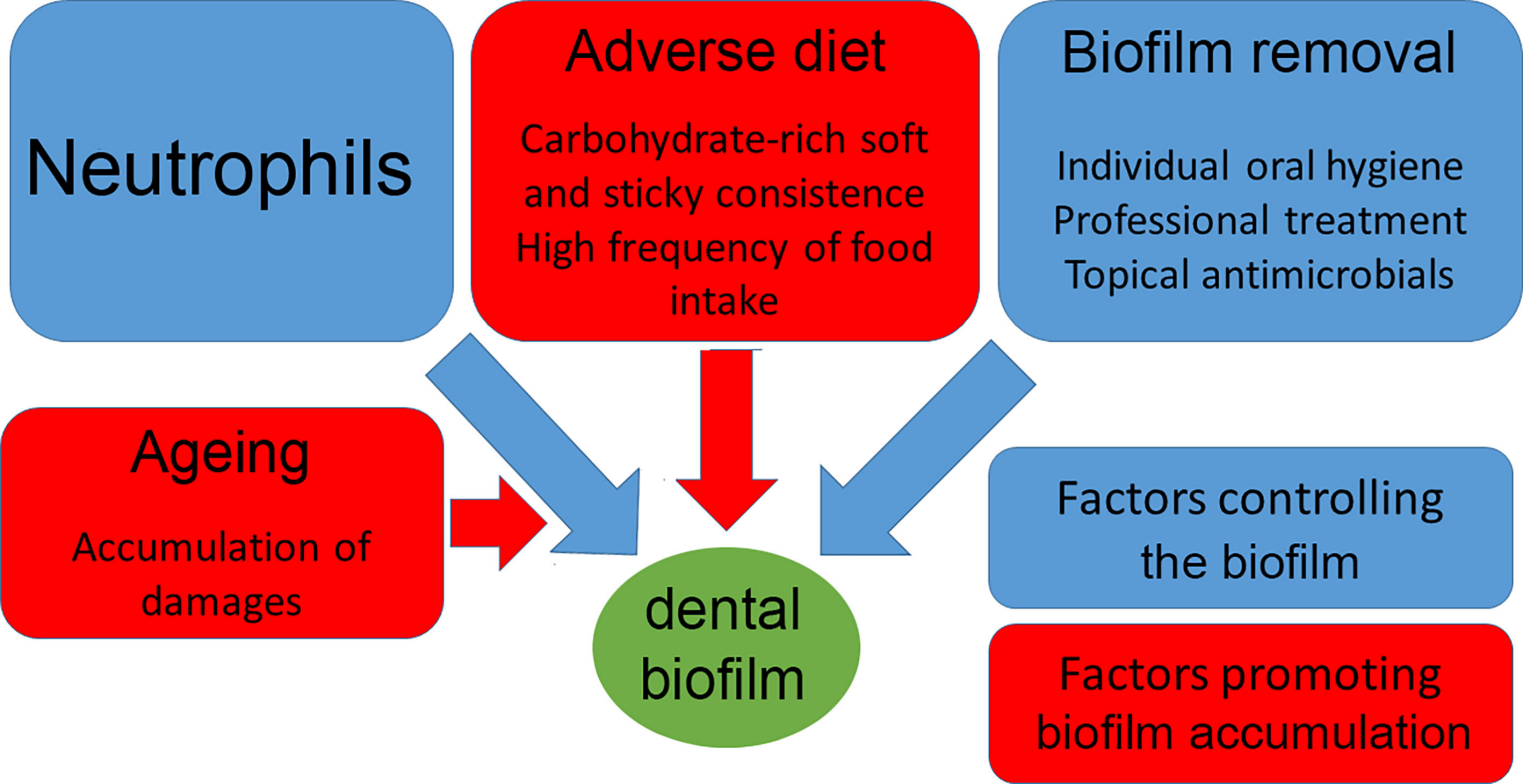
Relationship between host and dental biofilm.

Maintenance of tissue homeostasis is imperative to host survival. This fundamental process relies on a complex and coordinated set of innate and adaptive responses that calibrates responses against self, food, commensals, and pathogens (16). In health, homeostasis between gingiva and microbiota exists, i.e. the microbiota is controlled by the immunity (17) (**Figure 1**). In patients with neutropenia and defects of leukocyte adhesion early-onset periodontitis inevitably develops (18). Similarly, dysregulating the immunity *via* intra-gingival application of lipopolysaccharide results in experimental periodontitis without the contribution of any additional bacterial pathogens (19, 20). Patients with late-onset periodontitis have systemic low-grade inflammation and neutrophil hyper-responsiveness (21–25). Taken together, all forms of periodontitis are characterised with either neutrophil defects or dysregulated immunity, in particular neutrophil dysregulation. Periodontitis is not a consequence of basic alteration of the oral microbiota, but rather of the inability of the host immunity to resolve chronic inflammation (26, 27). The capacity of certain bacteria to act as a commensal or pathogen is highly dependent on the host immune conditions (28).

The development of a periodontal pocket marks the point of no return to homeostasis. The pocket reflects an impairment of the innate immunity to control the subgingival microbiota, as this pathological structure provides anaerobic conditions and mechanical protection for the accumulation of subgingival biofilm. It hampers the GCF flow through the prolonged duct-like crevice and facilitates the accumulation of PAMPs, proteases and is accompanied by an excess of reactive oxygen species (ROS). The periodontal inflammation can temporarily be suppressed by antimicrobials that reduce the dental biofilm (29), but not on permanent basis. Conservatively treated periodontal sites are subject to recolonization with a microbiota similar to that prior to therapy. The degree and speed of recolonization depends on the treatment protocol and the distribution patterns of periodontal microorganisms elsewhere in the oral cavity. It is further influenced by the quality of the patient’s oral hygiene (29). Nevertheless, the surgical elimination of periodontal pocket does not eliminate the proneness to relapse and does not substantially alter the microbiota (30). Albeit the sulcular microbiota in orally healthy subjects has been considered symbiotic, the differences between oral symbiotic and dysbiotic microbiota remain elusive (26, 27). The symbiosis between the microbiota and its mammalian host encompasses various forms of relationships, and how members of the microbiota interact with their host can be highly contextual with the same microbe developing as mutualist, commensal, or pathogens according to the genetic landscape and immunity of the host (16, 28). Increased inflammation in periodontitis is not associated with a distinct microbiome; it rather corresponds with higher dental biofilm biomass (31). Indeed, the higher biofilm biomass produces more PAMPs, hence a stronger inflammatory response and more host damages, as the lipopolysaccharide (LPS)-induced experimental periodontitis shows (19, 20). These findings support the alternative notion that the microbiota shift is due to dysregulated immunity (14, 15). Indeed, neutrophil hyper-responsiveness has been reported in late-onset periodontitis (21–25). The neutrophil hyper-responsiveness remains in edentulous patients with a history of late-onset periodontitis (21–23, 25), despite the disappearance of the bacteraemia after teeth exfoliation (32). Its control by the immune response is obviously insufficient in periodontitis-susceptible modern humans, who also have dissimilar diet and lifestyle than their pre-modern ancestors (33). The role of dental biofilm overgrowth is crucial (29), but it appears to be the consequence of dysregulated immunity (27); the aberrant responsiveness of neutrophils in periodontal disease supports this possibility (21–25). Lastly, the majority of modern people over 30 years remain periodontitis resistant (34).

Human beings are domestic beings seemingly eluding the “natural selection” and some of their evolutionary adaptations, like oral hygiene and professional dental treatment are artificial. The same applies to the pets. In comparison, advanced late-onset periodontitis has not been reported in wild-living Mammalia. Periodontitis completely lacks in rhinoceros (35), or is rare in marmosets (36). In wild-living apes, only occasional and mild forms of periodontitis have been reported (37).

## Dental Biofilm

### Biofilm Basics

Biofilms are aggregates of interface-associated sessile bacteria, Candida, and viruses (38), all embedded within a matrix of extracellular polymeric substances (EPS) (39–41). Horizontal transfer of EPS genes (42) plays an important role in the multi-species consortia of dental biofilm (43, 44). Each oral biofilm consists of five main components: (I) bacteria, (II) bacterial membrane vesicles (BMVs), (III) macromolecules like proteases and toxins, (IV) immune cell and their remnants, all encapsulated by (V) EPS. The biofilm EPS offers protection from the surrounding environment and provides certain advantages to the embedded sessile bacterial community that planktonic bacteria do not possess (45). Biofilms go through a life cycle of planktonic cell attachment to an interface, micro-colony formation, biofilm maturation, and finally dissemination (46). Each phase of this circle is characterised by distinct bacterial phenotype. Thus, the biofilm is a complex microbial community characterised by attributes not seen in planktonic bacteria: (I) primitive homeostasis and metabolic cooperativity (47) (II) cell-to-cell signalling (39) (III) essentially higher resistance to antibiotics (48) (IV) dissemination by dispersion (49) (V) detachment of bacteria (50–52) and last not least (VI) resistance to the host defence (40). Thus, many oral pathogens are protected from crevicular neutrophils within the bulky dental biofilm and can rely on nutrition from ingredients of GCF diffusing through the EPS. Thereby, the gingivitis is beneficial to the biofilm, as the first host response of gingivitis involves increased flow rate of the GCF (53). The role of dental biofilm is beyond doubt, as its removal efficiently prevents gingivitis and periodontitis. Importantly, even a short-term biofilm accumulation is sufficient to induce gingival inflammation (53). Dental biofilm is a *conditio sine qua non* for biofilm-induced periodontitis (29). In individuals without gingivitis and periodontitis, biofilm might be partly removed *via* mastication, but the artificial biofilm destruction, i.e. teeth brushing, is by far more efficient (54). The abundance of carbohydrates and the soft consistency of the diet in post-industrial revolution societies lead to biofilm overgrowth as mechanical biofilm destruction is insufficient and carbohydrates foster biofilm accumulation (55). Surviving some biofilm parts enables the biofilm maturing. This is characterised by phenotype transition into the so-called state of persisters, bacteria highly resistant to antibiotics and neutrophil killing (see § 3.3). Both persisters and EPS hinder neutrophil killing and prevent destruction of the biofilm by crevicular neutrophils (56). Modern humans attempt to compensate for this failed biofilm removal by targeting dental biofilm through oral hygiene and professional treatment. These are the new evolutionary strategies of the post-industrial age. However, only susceptible individuals are affected by periodontal disease; most humans, i.e. nearly 60%, are resistant and remain periodontally healthy lifelong (34). This fact suggests the existence of unknown immune mechanisms, able to completely protect orally healthy individuals from oral microbiota.

### Heritability of Oral Microbiota and Shift Due to Diet

Oral microbiota is primarily inherited from caregivers (17). Genetic studies in twins have shown that host-related microbial communities depend on both host genetics and environmental factors (57). Oral microbiome similarity increases with shared host genotypes. Highly heritable oral taxa have been identified, although most of the variation of the oral microbiome has been determined by environmental factors (57). These findings indicate that the hosts control almost half of their oral microbiota. The remaining half differs due to differences in diet, lifestyle and environment (29) and hence are “independent” of the host genotype. From the microbiologic perspective, the environment shapes the microbiota (17). The environment of supragingival biofilm microbiota is determined by both host and diet. That of subgingival biofilm predominantly by host, as the subgingival effects of diet are minor However, a discrimination between supragingival and subgingival biofilm has been rarely reported in oral microbiome studies (31). Subgingival biofilm is found in the gingival sulcus of people without and in the pocket of people with periodontal disease. Discrimination between supragingival and subgingival biofilm in fossils of individuals without periodontitis is not possible and only partly feasible in living animals for technical reasons. The fact that no discrimination has been reported in studies on fossils and animals (33, 58) strongly suggests that the examined biofilm was either supragingival or mixed, thus limiting the usefulness of these studies on oral microbiome concerning periodontitis.

With the establishment of the agricultural and industrial lifestyle and diet alteration, there was a shift in the oral microbiota and the number of oral pathogens increased, especially of *Porphyromonas gingivalis (P. gingivalis)* (33). However, not only the carbohydrate-rich Neolithic diet, but also the low chewing resistance leads to the formation of biofilm. The importance of the mechanical destruction of dental biofilm in modern humans is beyond doubt and they solve this problem artificially, at least in part, by brushing teeth, flossing and professional oral hygiene (59).

### Planktonic Bacteria and Biofilm Differ

A fraction of biofilm bacteria evolves into persister cells that are genetically nearly identical, but phenotypically distinct from their parent cells. Persisters are metabolically inert, replicate slowly, modulate the toxin-antitoxin system, upregulate DNA repair and anti-oxidative machinery, have enhanced phosphate metabolism, and exhibit unresponsiveness towards minimal inhibitory concentrations of antibiotics (60). Drug treatment normally kills planktonic cells and the majority of biofilm cells. Nevertheless, EPS and drug tolerant persisters remains unharmed. The latter repopulate the biofilm, disseminate into planktonic forms and start a new cycle of biofilm development (60–62). This perpetuates diseases caused by biofilm forming pathogenic microorganisms. For this reason, the biofilm is considered of “pseudo-organismic nature” (27). Therefore, new strategies aiming at EPS destruction have been introduced (41).

## Immunity Dysregulation as Instigator of Periodontitis

Many features of periodontitis appear to be unrelated to the composition of oral microbiota. They can be used to examine to what extent the oral microbiota determines development and maintenance of periodontitis.

### Heritability of Periodontitis: Dysbiosis as a Consequence of Immune Deficiency

The genetic backdrop of all forms of early-onset periodontitis is based on inborn neutrophil defects (18, 63) or on other inborn genetic defects leading to neutrophil activation (64). The concomitant dysbiosis in early-onset periodontitis is hence a consequence of inborn immune deficiency. The genetic predisposition of late-onset periodontitis is beyond doubt (65). However, the association of genetic polymorphisms in PAMP-sensing and PAMP-signalling genes with the microbiota composition has been detailed studied only in gut. Importantly, host cells sense bacteria *via* their PAMPs by pattern recognition receptors (PRRs) (66–73). PRRs include several families of receptors: toll-like receptors (TLRs), nucleotide-binding oligomerisation-like (NOD-like) receptors, RIG-I-like receptors, and C-type lectin receptors. Thus, gut dysbiosis has been demonstrated in studies on knockout TLRs in mice (67). Nod2-deficient mice showed an increased load of commensal resident bacteria, a reduced ability to prevent intestinal colonisation by pathogenic bacteria (69, 70), and an increased susceptibility to bacterial infections (71). Inflammasome-deficient mice have an impaired host/microbiome interaction causing an increased intestinal inflammation (66, 72, 73). A locus containing the Irak4 gene, a kinase that activates the nuclear factor-kB pathway in TLR-and T cell receptor-signalling pathways is associated with certain bacterial species (74). Several bacteria are associated with the locus that contains the Irak3 gene, another regulator of TLR-signalling pathway (75). Consistent findings have been observed for the association of PRR genes with microbiome composition and microbiome-associated disease (76). So, single-nucleotide polymorphisms in the NOD1 gene are associated with bacterial pathways and gene groups specific for *E. coli* (68). Genetic variants in NOD2 are strongly associated with Crohn’s disease, an inflammatory condition of the gut associated with dysbiosis (77, 78)]. Carriership of the NOD2 genetic risk for Crohn’s disease is associated with an increased relative abundance of Enterobacteriaceae (77). An increased risk of periodontitis among patients with Crohn’s disease has been well established (63, 78).

The host genetic backdrop of dysbiosis indicates that it is in many cases a consequence of immune defects respectively dysregulation. In addition, the inability to transfer oral pathogens within the family (79) or the merely transient effect of a single oral microbiota transplant in dogs (80), supports the concept that the dysbiosis of dental biofilm is a result of the dysregulated host immunity and not vice versa. Neither microbiota transplantation (80) nor probiotics have any effect on periodontitis (81) and gingival inflammation (82).

### Ageing. Periodontitis as a Common Mammalian Age-Related Disease

Postnatal development of an individual is followed by a “middle” period of relative stability, when changes in physical and cognitive function are small and only detectable by very sensitive tools or challenging tests. During that period, most individuals in the population are free of diseases (83). This also applies to people who develop late-onset periodontitis over the age of 30 (2–4). Underneath this apparent stability, several compensatory and homeostatic mechanisms continuously operate to preserve the biochemical balance and prevent phenotypic derangements, as well as functional decline (84). These mechanisms are initially effective and provide a robust homeostasis, but start to fade later in life. Ageing is a complex process involving various mechanisms that lead to the accumulation of subcellular, cellular and intercellular damages as well as other age-related deleterious changes, together representing the organisms’ “deleteriomes” (85). Unrepaired damage accumulates beyond the functional threshold (84). On the molecular level, the most precise biomarker of ageing is based on DNA methylation profiling and is known as the “epigenetic clock” (86).

Periodontitis is a common mammalian disease affecting humans, non-human primates (87), ruminants (88, 89), rodents (11, 90), and pets (91, 92). Periodontitis has been reported in Palaeolithic, Mesolithic, Neolithic and post-industrial revolution humans (93). An important question is, whether the prevalence of periodontitis increases in Neolithic and post-industrial revolution or just the average human longevity. This question cannot be answered without doubt. The synchronous increase of average human longevity and the accompanying increase in prevalence of periodontitis creates the impression of genuine increase in prevalence in post-industrial humans. This has been mainly explained as a consequence of altered diet and lifestyle (33). However, the accumulation of somatic mutations dysregulates the immunity and results in a plethora of age-related diseases (94), which become flashier with the lifespan increase in both humans (34) and domestic mammalians (90) during the post-industrial revolution. The prevalence increase of late-onset periodontitis underlies the same kinetics. It can be deduced that anti-aging therapies should alleviate periodontal disease. Indeed, the good responsiveness of periodontitis to anti-ageing therapies (11–13) scrutinise the role of dysbiosis in periodontitis and highlights the role of dysregulated immunity. On cellular level, *Porphyromonas gingivalis* causes *in vitro* cell senescence, which is reversed by anti-ageing treatment (95).

### Dysbiosis and Immunity

The capacity of certain bacteria to act as a commensal or pathogen is highly dependent on the host immune conditions, genetic predispositions, and coinfections (28). The host poses a complex regulatory system, involving epithelial cells, IgA, AMPs, and an array of innate and adaptive immune cells to control the composition and distribution of the microbiota (16). Thus, periodontitis heritability, ageing relatedness and neutrophil hyper-responsiveness (10) are consequences of immunity dysregulation. This underscores the role of immunity and enables a new perspective on periodontal disease, independently of the dysbiosis tenet. The common definition of dysbiosis is an imbalance between beneficial and harmful microorganisms (96–98). This requires the existence of “healthy” microbiota in healthy individuals that can be used as a reference pattern for differentiation between health and disease. However, a “healthy” microbiota pattern has never been established (99, 100). Especially in oral microbiota, where the diversity is very high (101), and strongly differs depending on culture, diets, regional differences, age, social state etc. The lack of “healthy” oral microbiota relativises the pathogenic role of oral dysbiosis. Recently, the dysbiosis in general (but not the oral one) has been hypothesised to be a consequence of the dysregulated immunity (14, 15, 102), and a new environmental concept of microbiota regulation by the host as its environment has been established (16, 17, 28). In knocked out CXCR2^-/-^ mice, which are characterised by the absence of gingival neutrophils, the oral microbiome undergoes a significant shift in total load and composition as compared to that of wild type CXCR2^+/+^ mice with normal levels of neutrophil recruitment into the gingival tissues. This dysbiosis in CXCR2^-/-^ mice is accompanied by a significant increase in periodontal bone pathology (103). However, transfer of the oral microbiome of CXCR2^-/-^ mice into germ free CXCR2^+/+^ mice led to restoration of the microbiome to the wild type CXCR2^+/+^ composition and the absence of pathology. These data demonstrate that the composition of the oral microbiome is governed to a significant extent by the genetically determined immunity of the host organism (103).

## Dysregulated Immunity

### Neutrophil Functions in Gingiva

Neutrophils play a central role in the control of bacterial infections. Neutrophils are also the effector immune cells responsible for the antimicrobial defence in the gingiva and the first defenders to face the bacterial invasion. Thus, the tissue neutrophil density increases at least 150-fold in the first 4 h after intradermal inoculation of healthy rabbits with *E. coli*, since a certain neutrophil density is required to counter bacterial invasion (104). The indispensable role of neutrophils in periodontal health is evident from development of early-onset periodontitis in patients with neutropenia and with defects of leukocyte adhesion (18). Neutrophils do not recognise individual pathogens or pathogen species, but just danger signals, (I) chemokines, (II) cytokines, (III) immune complexes, (IV) PAMPs, (V) damage-associated molecular patterns (DAMPs), (VI) C3a or 5a, and (VII) complement C3 and C4 and their derivatives (105). Intra-gingivally injecting LPS is sufficient to cause experimental periodontitis and is routinely used as animal model (19, 20). Vice versa to the surplus of PAMPs, the increased PMN responsiveness characteristic for late-onset periodontitis (21–24) may effectuate the same result. Thus, neutrophils can turn from bacterial defender into tissue devastators independently from the bacterial challenge (105, 106). In particular, components of exaggerated NETs harm and even kill epithelial cells (107) and promote tissue damage (108, 109).

### Trained Immunity: Neutrophil Hyper-Responsiveness and NET Aggregation

The neutrophil hyper-responsiveness in late-onset periodontitis is also an aspect of dysregulated immunity (21–25); it persists in edentulous patients with a history of periodontitis (21–23, 25). Certain microbial challenges promote the response of myeloid cell populations to subsequent infections either with the same or with other pathogens. This phenomenon involves changes in the cell epigenetic and transcription, and is referred to as ‘‘trained immunity’’ (110). It acts *via* modulation of hematopoietic stem and progenitor cells (HSPCs). A main driver of modulation is the sustained low level transfer of lipopolysaccharides from the periodontal pocket to the peripheral blood. Dysregulated trained immunity misleads the neutrophils to a non-resolving inflammatory state with elevated and reduced levels of inflammatory and homeostatic mediators, respectively (111). In general, trained neutrophils are prone to increased NET formation (112, 113). The neutrophil hyper-response aims to destroy the pocket pathogens, but they appear to be resistant to NET killing (114). So, bystander damages, due to the surplus of NET proteases and histones, are responsible for lessening the epithelial barrier and formation of ulceration (see §4.4). Both sorts of epithelial damage compromise the gingiva defence. Thus, the exaggerated NET formation in late-onset periodontitis (115) may also be a result of trained immunity. Trained immunity gives neutrophils a partial “autonomy” that does not underlie the direct control of adaptive immunity (10).

### NET Response in Periodontitis

Dental biofilms communicate with the crevicular neutrophils *via* soluble excretions of dental biofilms, mostly PAMPs, recognised by neutrophil surface receptors (116). However, in periodontitis neutrophil toll-like receptors (TLRs) may be degraded by the increased concentrations of crevicular neutrophil proteases (117–121). Interestingly, when neutrophils are stimulated *in vitro* with oral pathogens, TLR inhibitors have no effect on ROS and NET release (114); this indicates that TLRs are not involved in the activation of crevicular neutrophil. An alternative bacterial recognition takes place *via* outer membrane vesicles (OMVs) (122). These are endocytosed by neutrophils and activate caspase-4/11 (123). Gram negative bacteria prevail in subgingival biofilms. Thus, the main share of BMVs from dental biofilm in periodontitis are OMVs that are heavily loaded with LPS (122). OMVs are released into the GCF by bacterial biofilms during normal cell growth without affecting cell viability; but growth conditions have a profound effect on the release of OMVs (124, 125). Two main mechanisms are responsible for bacterial and OMV dissemination from biofilms: (I) bacterial dispersion, an active process controlled by various biofilm-intrinsic mechanisms, like *quorum sensing* (49) and (II) detachment, a passive process driven by mechanical forces (46). During mastication and hygiene procedures, subgingival dental biofilms are exposed by a pump-like action of the periodontal pocket. It is accompanied by bacterial translocation, a clear indication of a biofilm detachment (50–52). Indeed, free LPS is recognised by membrane-borne TLR4 and induces NET formation *via* the MEK/ERK pathway (126); this is similar to the action of PMA (127) and activates several transcriptional nuclear factors. OMVs function as vehicles that deliver LPS into the cytosol. When endocytosed, OMVs release LPS from the early endosomal compartments into the cytosol (122). The host is thus capable of TLR4-independent cytosolic recognition of LPS (128, 129). Inflammatory caspases, namely murine caspase-11 and human caspase-4 and caspase-5 serve as receptors for cytosolic LPS (130). The latter also induces caspase 4/5/11-dependent cleavage of gasdermin D (GSDMD) and thus promotes suicidal NET formation, whereas caspase 1 is not activated (123). Otherwise, NET induced by canonical stimulants proceed caspases-independently but share the morphological features of NET formation induced by caspase-4/5/11/GSDMD signalling (123).

Another possibility to trigger NET formation when TLRs are proteolytically degraded involves the cleavage of the protease activated receptor (PAR2) on neutrophils surfaces, e.g. by gingipain. Importantly, NETs formed in this way are deficient in antibacterial activity (131), hence it is evident that the PAR2-based responses do not orchestrate the host’s defence but drive gingival damage (131).

### Neutrophil-Induced Gingival Damages

Hyper-responsive neutrophils and in particular exaggerated NET formation cause tissue damage (108). Abundant crevicular neutrophils and NETs overload the pocket with neutrophil-derived proteases (118, 120, 132) and cause epitheliopathy *via* Oncostatin M (133). This correlates with the epithelial ulceration in periodontitis (5, 134). NET-derived components such as histones (107, 135–137) and myeloperoxidase (MPO) (107) are cytotoxic to epithelial cells; neutrophil proteases damage and even kill epithelial cells. High NET levels reportedly suppress keratinocyte proliferation, delay wound closure (138, 139) and chronifies ulcers. In contrast, aggNETs proteolytically inactivate several soluble pro-inflammatory mediators over time (140). Neutrophil activation due to plasminogen (Plg) deficiency causes periodontitis in both humans (known as ligneous periodontitis) (141) and Plg^-/-^ mice (64). The neutrophil activation in Plg deficiency is effected *via* fibrin polymer binding motif recognisable by the integrin αmβ2 (CD11b/CD18) (142) and results in exaggerated NET formation in Plg^-/-^ mice. The exaggerated NET formation effectuates heavy periodontitis, which can be suppressed by DNase I in a mouse model (64). Exaggerated NET formation is concomitant with heavy purulent periodontitis (6).

Gingival homeostasis does not require oxidative burst, as periodontitis occasionally occurs in patients with chronic granulomatous disease (CGD), a rare primary immunodeficiency that affects the innate immune system. It is caused by mutations in any of the four genes encoding the subunits of the superoxide generating phagocyte NADPH oxidase; CGD displays no or very low levels of enzyme activity (143). Some isolated cases of periodontitis have been reported in CGD patients (144–146). A survey on 368 CGD patients has reported just nine cases of gingivitis or periodontitis (147). However, excess of ROS characterises periodontitis-related neutrophil hyper-responsiveness (21–25). Consequently, the deleterious effects of ROS on host tissues (148) are boosted in periodontitis. NETs entrap oral bacteria, but do not kill them (114). Thus, the surplus of both ROS and proteases in periodontitis harms the host, a clear indication of dysregulated immunity.

### Neutrophil Orchestration by Gingiva and the Adaptive Immunity

Oral epithelial cells are sentinel cells provided with a multitude of PRRs and upon PAMP stimulation produce interleukins (ILs), in particular IL-8 (149). IL-8 is mostly recognised *via* the neutrophil chemokine receptor CXCR2, which play a crucial role for the neutrophil recruitment into periodontal crevice (150). After penetrating the epithelial barrier, lipopolysaccharides mount a strong inflammatory response of gingival fibroblasts *via* their surface-expressed TLR-4 (151). In periodontitis, there is extensive inferred communication between stromal and immune cells (152). Of interest and consistent with pathways upregulated in disease, stromal and epithelial cells appeared to promote adhesion of immune cells, while fibroblasts displayed a potential toward recruitment of inflammatory cells. Gene-expression signatures indicate an active role for stromal cells in the recruitment of immune cells to the site of disease (152). Fibroblasts are particularly transcriptionally active in the production of chemokines. Fibroblasts expressed a broad array of chemokine ligands exclusive in their potential to recruit neutrophils (CXCL1, 2, 5, 8) as well as chemokines with the potential of recruiting several types of leukocytes, e.g. CXCL12, CXCL13, CCL19. Taken together, these data suggest that stromal cells utilize intercellular signalling to drive immune cell recruitment and tissue transmigration in periodontitis (152) (152).Though the gingival inflammatory response is dominated by neutrophils (153), the entire immune response is involved. Within the crevice, IgA binds to neutrophil Fc-alpha receptors; thus the adaptive immunity guides the neutrophil response (154–156). Once the adaptive immunity has developed, the neutrophil response in periodontitis is orchestrated by Th17 cells (32, 157).

## Conclusion

Dental biofilms are aggregates of tooth surface-associated sessile bacteria. They are characterised by phenotype transition of a few bacteria into the so-called state of persisters, cells highly resistant to antibiotics and neutrophil killing. Both persisters and EPS hinder neutrophil killing and prevent destruction of the subgingival biofilm by crevicular neutrophils. Each microbiota depends on environmental factors, so host-related microbiota depends on host genetics and immunity. Ageing of persons developing late-onset periodontitis is a complex process involving various mechanisms that lead to the accumulation of subcellular, cellular, intercellular and other deleterious changes of immunity. Due to neutrophil defects or immunity dysregulation, both a shift in oral microbiota and periodontal damage occur, the homeostasis between host and microbiota is disbalanced and the latter is no more under the complete control of immunity. The inability of immunity to control the biofilm results in biofilm overgrowth and increased number of periodontal pathogens. As a consequence of dysregulated trained immunity, the neutrophils become hyper-responsive. The neutrophil hyper-response is aimed to destroy the pocket pathogens, but they appear to be resistant to NET killing, so gingiva damage occurs, due to the excess of NET proteases and histones. The last two are blameable for damages of epithelial barrier, its penetration by bacteria and PAMPs as well as the stimulation of Th17 cells, resulting in further neutrophil activation and host tissue damage.

## Author Contributions

Conceptualization and writing—original draft preparation, LV. Writing—review and editing, LV, MH, MHa, JK, LM, SJ, and CS. Funding acquisition, MHa and MH. All authors have read and agreed to the published version of the manuscript.

## Funding

This research was supported by the German Research Foundation (DFG) Grants No. 2886 PANDORA B3; SCHA 2040/1-1; CRC1181(C03); TRR241(B04), by the EU H2020-FETOPEN-2018-2019-2020-01; 861878 “NeutroCure”, and by the Volkswagen-Stiftung (Grant 97744).

## Conflict of Interest

The authors declare that the research was conducted in the absence of any commercial or financial relationships that could be construed as a potential conflict of interest.

## Publisher’s Note

All claims expressed in this article are solely those of the authors and do not necessarily represent those of their affiliated organizations, or those of the publisher, the editors and the reviewers. Any product that may be evaluated in this article, or claim that may be made by its manufacturer, is not guaranteed or endorsed by the publisher.

## Abbreviations

BMVs, bacterial membrane vesicles; CGD, chronic granulomatous disease; DAMPs, damage-associated molecular pattern; DOAJ, directory of open access journals; EPS, extracellular polymeric substances; FcR, Fc receptor; GCF, gingival crevicular fluid; NADPH, Nicotinamide adenine dinucleotide phosphate; HSPC, hematopoietic stem and progenitor cells; IgA, Immunoglobulin A; LPS, lipopolysaccharide; NETs, neutrophil extracellular traps; NOD-like, nucleotide-binding oligomerisation-like; OMVs, outer membrane vesicles; PAMPs, pathogen-associated molecular patterns; PAR2, protease activated receptor 2; Plg, plasminogen; PRRs, pattern recognition receptors; RIG-I, retinoic acid-inducible gene I; TLR, toll-like receptor; TNF, tumour necrosis factor.
